# Efficacy of *Ficus tikoua* Bur. extract in ethylene glycol-induced urolithiasis model in SD rats

**DOI:** 10.3389/fphar.2022.974947

**Published:** 2022-08-29

**Authors:** Arina V. Bervinova, Viktor A. Palikov, Evgeny S. Mikhailov, Yulia A. Palikova, Natalya A. Borozdina, Vitaly A. Kazakov, Pavel A. Rudenko, Elena A. Tukhovskaya, Igor A. Dyachenko, Gulsara A. Slashcheva, Natalya A. Goryacheva, Elena S. Sadovnikova, Irina N. Kravchenko, Elena A. Kalabina, Maksim V. Shinelev, Peng Wu, Arkady N. Murashev

**Affiliations:** ^1^ Branch of Shemyakin and Ovchinnikov Institute of Bioorganic Chemistry, Russian Academy of Sciences, Pushchino, Russia; ^2^ Pushchino State Institute of Natural Sciences, Pushchino, Russia; ^3^ Chengdu Sino PharmTech Co., Ltd., Chengdu, China

**Keywords:** urolithiasis, Ficus tikoua Bur., ethylene glycol, Cystone^®^, SD rats

## Abstract

The development of new herbal preparations for the treatment of urolithiasis is an urgent task of medical science. Ficus have attracted the attention of pharmacologists due to a wide range of biological properties, including antioxidant, anti-inflammatory, antibacterial and antifungal activity. We studied the effectiveness of *Ficus tikoua* Bur. in SD rats in which urolithiasis was induced by 6 weeks of oral administration of ethylene glycol 0.5% *ad libitum* instead of drinking water. Administration of the extract of *Ficus tikoua* Bur., as well as comparative drug Cystone^®^ after modeling of urolithiasis lead to the restoration of diuresis and the concentration of inorganic phosphates starting from the 6th week of the experiment. The use of the *Ficus tikoua* Bur. extract for 6 weeks, both during the modeling of urolithiasis and during the recovery period, led to the restoration of the percentage of lymphocytes in the blood, content of sodium, chlorine and inorganic phosphates in the blood to the control level. Thus, the extract of *Ficus tikoua* Bur. seems to be a promising drug for effective treatment of the initial stages of the development of urolithiasis.

## 1 Introduction

Urolithiasis is widespread among people - up to 9% in a representative sample of the general population (Croppi et al., 2012; Scales et al., 2012; Siener, 2021). After the development of a kidney stone, the recurrence rate is 14% at 1 year, 35% at 5 years, and 52% at 10 years. The incidence among the world population as a whole is approximately 1 in 1,000 adults per year (Vyas and Argal, 2013; Eisner and Goldfarb, 2014; Rule et al., 2014; Ferraro et al., 2017). Thus, primary and secondary prevention of urinary tract urolithiasis is an urgent medical problem. Diet and lifestyle changes are an important strategy for preventing kidney stone recurrence. Factors influencing the formation of kidney stones are modifiable and are associated with lifestyle and diet (Frassetto and Kohlstadt, 2011; Ferraro et al., 2017).

There are various treatments that are used for preventive therapy, but there is no drug that can dissolve a kidney stone. Surgical stone removal, extracorporeal shock wave lithotripsy, and percutaneous nephrolithotomy can cause hemorrhage, hypertension, tubular necrosis, and subsequent renal fibrosis (GhelaniH et al., 2016). Herbal preparations have a multifaceted effect, including the prevention of stone formation, the dissolution of calculi, the facilitation of the independent discharge of calculi and their fragments after disintegration, the prevention of exacerbations of chronic infectious and inflammatory diseases of the urinary tract.

The development of a herbal preparation for the treatment of urolithiasis is currently one of the urgent tasks of medicine and pharmacology (Bagcı, 2000; Fabricant and Farnsworth, 2001; Muthu et al., 2006). An example of such a drug is Cystone^®^, which contains components of plant origin. Cystone^®^ is often used as a conservative treatment for urolithiasis and is used as a comparator when studying the effectiveness of new drugs. Cystone^®^ regulates the crystal-colloid balance in dysmetabolic nephropathy, reduces the concentration of oxalic acid, calcium and hydroxyproline in the urine, which contribute to the formation of stones (Alenzi et al., 2017). Cystone^®^ prevents the accumulation of particles around the core of the stone, which prevents its further growth, relaxes the smooth muscles of the urinary tract, thereby stimulating diuresis and promotes the removal of oxalate and phosphate salts, uric acid and small stones from the urinary tract. Cystone^®^ also has a bacteriostatic and bactericidal effect, especially against Gram-negative bacteria (Erickson et al., 2011; Shah et al., 2012; Bawari et al., 2020).

*Ficus tikoua* Bur., a plant belonging to the Moraceae family, is widely distributed in southern China, India, Vietnam and Laos and has long been used in traditional folk medicine to treat a number of diseases, such as chronic bronchitis, diarrhea, dysentery, mastadenitis, rheumatism, edema, impetigo, cough caused by fever of the lungs (Guan et al., 2007; Ahmed and Urooj, 2010; Wei et al., 2012). *Ficus tikoua* Bur. extract contains several ingredients that work synergistically. Previous studies have shown that phenylpropanoids, flavonoids, coumarins, lignans, chromones, triterpenoids, sesquiterpenoids, and alkaloids are the most common secondary metabolites isolated from the genus Ficus (Park et al., 2001; Chiang et al., 2005; Karadi et al., 2006; Taiwo et al., 2006; Damu et al., 2009; Chen et al., 2010; Donfack et al., 2010; Muanda et al., 2010; Thakare et al., 2010; Wang et al., 2010).

To study the effectiveness of drugs intended for the treatment of urolithiasis, the ethylene glycol model of urolithiasis is most often used. The ethylene glycol model reliably reproduces human nephrolithiasis that occurs against the background of primary hyperoxaluria. There are many options for modeling urolithiasis using ethylene glycol at concentrations from 0.5 to 1% (Khan, 2004; Khan, 2006; Laroubi et al., 2007; Corley et al., 2008; Tzou David et al., 2016). However, the use of 1% ethylene glycol, as well as its combination with ammonium chloride, leads to multiple organ failure in rats and the development of acute renal failure in 3–4 weeks of modeling, which does not correspond to the chronic course of urolithiasis in humans (Mann et al., 2012). In this regard, in our study, it was decided to induce urolithiasis with 0.5% ethylene glycol and, in this model, to study the effectiveness of oral administration of the *Ficus tikoua* Bur. extract in SD rats. The aim of our study was to study the activity of the *Ficus tikoua* Bur. extract in a model of urolithiasis in SD rats.

## 2 Materials and methods

### 2.1 Animals

84 mature specific pathogen free (SPF) male SD rats, 10–12 weeks old were obtained from the Pushchino nursery of laboratory animals (Pushchino, Russia). All procedures and manipulations with animals were approved by the Committee for Control over Care and Use of Laboratory Animals of BIBCh RAS (IACUC) (protocol number 730/20 from 18.02.2020) and were carried out in accordance with the EU Directive 2010/63/EU. The Biological Testing Laboratory of Branch of Shemyakin and Ovchinnikov Institute of Bioorganic Chemistry, Pushchino, Moscow Region, Russia, where experiments were carried out, has AAALAC accreditation (https://www.aaalac.org/accreditation-program/directory/directory-of-accredited-organizations-search-result/?nocache=1#home_acc_dir_search, last renewal of accreditation on 17 November 2017, last access at 08.06.2022). After receiving from the nursery, the animals underwent adaptation within 7 days. During the study, the animals were kept under controlled environmental conditions in a barrier zone with a “clean” and “dirty” corridor system with controlled environmental conditions (temperature 20–24°C, relative humidity 30–55%, 12-h light cycle). 08:00–20:00 - “day”, 20:00–08:00 - “night”, 10-fold change in air volume in the room per hour). SNIFF RI/M-H V1534-30 complete granular rodent food was autoclaved and fed *ad libitum*.

### 2.2 Experimental design

#### 2.2.1 Groups and doses

A total of 84 mature male SD rats were used. Animals were divided into the following five groups: control group - animals received *ad libitum* standard water filtered by the MilliRO Millipore system (Control, *n* = 24); group 2- animals received *ad libitum* instead of drinking water ethylene glycol 0.5% (EG 0.5%, *n* = 24); group 3 - urolithiasis was simulated in animals by replacing drinking with 0.5% EG for 6 weeks with the simultaneous administration of *Ficus tikoua* Bur. extract at a dose of 250 mg/kg daily for 6 weeks, starting from the 1st day of the study (EG 0.5% + Ficus 1, *n* = 12); group 4 - urolithiasis was simulated in animals by replacing drinking with 0.5% EG for 6 weeks after which the animals received *Ficus tikoua* Bur. extract at a dose of 250 mg/kg daily for 6 weeks, starting from the 7th week of the study, that is, after the withdrawal of 0.5% EG (EG 0.5% + Ficus 2, *n* = 12); group 5–urolithiasis was simulated in animals by replacing drinking with 0.5% EG for 6 weeks after which they received Cystone^®^ at a dose of 150 mg/kg daily for 6 weeks, starting from the 7th week of the study (EG 0.5% + Cystone^®^, *n* = 12) (Table 1).

**TABLE 1:** Groups and doses.

Group	Urolithiasis simulation	Drug	Dose	Route and scheme of administration	Number of animals
1	Vehicle (water) for 6 weeks	—	—	—	24
2	EG 0.5%as a drink in a drinking bottle for 6 weeks instead of water	—	—	—	24
3	EG 0.5% as a drink in a drinking bottle for 6 weeks instead of water	*Ficus tikoua* Bur. extract	250 mg/kg	Gavage 5 ml/kg simultaneously with EG 0.5% for 6 weeks from the first day of study	12
4	EG 0.5% as a drink in a drinking bottle for 6 weeks instead of water	*Ficus tikoua* Bur. extract	250 mg/kg	Gavage 5 ml/kg beginning from 7th week of study (after the withdrawal of EG0.5%)	12
5	EG 0.5% as a drink in a drinking bottle for 6 weeks instead of water	Cystone^®^	150 mg/kg	Gavage 5 ml/kg beginning from 7th week of study (after the withdrawal of EG0.5%)	12

#### 2.2.2 Modeling of urolithiasis

Beginning on day 1 of the study, for 6 weeks, the animals of groups 2–5 were given a solution of 0.5% ethylene glycol (EG 0.5%) *ad libitum* instead of drinking in drinking bottles. At the end of 6 weeks of EG 0.5% supplementation, animals received MilliRO Millipore filtered water *ad libitum*, in drinking bottles, for a subsequent 6 weeks of withdrawal.

#### 2.2.3 Drugs

Test article–*Ficus tikoua* Bur. extract, Sihcuan Senke Pharmaceutical Co., Ltd., China; comparative article - Cystone^®^, Himalaya Drug Company, India; article for simulation of urolithiasis–ethylenglicol, Russia.

For the study, *Ficus tikoua* Bur. extract was provided by Sihcuan Senke Pharmaceutical Co., Ltd., China. In the production of the extract, the stems of *Ficus tikoua* Bur. were harvested and cleaned, then crushed and soaked. The extraction was carried out by the methods of flow, boiling and reflux. Next, the concentration and drying of the finished extract was carried out. To prepare doses for administration, the required amount of extract was weighed, the required amount of water was added, and a suspension was obtained by stirring the mixture on a magnetic stirrer. Dosing to animals was carried out with constant stirring of the suspension using a magnetic stirrer to evenly distribute the extract in the suspension.

#### 2.2.4 Microscopic analysis of urine

For all animals at 3, 6, 9 and 12 weeks after the start of the study, microscopic analysis of the first portion of urine collected when animals were placed in a metabolic cage for a period of 24 h were performed. Microscopic assessment of inorganic sediment and differential count of urine crystals (phosphate and oxalate crystals) were performed. For this purpose, urine samples of 0.5 ml were centrifuged for 15 s at 3,000 rpm on a MiniSpin centrifuge (Eppendorf) and the sediment was analyzed on an Olympus CX 21 microscope at magnification ×10. Photomicrographs were taken of each urine sample. The micrographs were used to estimate the number of crystals.

#### 2.2.5 General urinalysis

A general urinalysis was performed by applying a urine sample to Aution Sticks 10 EA test strips (ARKRAY Factory, Inc., Japan), which were placed for analysis in a Pocket Chem PU-4010 semi-automatic urine analyzer (ARKRAY Factory, Inc., Japan).

#### 2.2.6 Blood sampling

Blood was taken during euthanasia from the inferior vena cava in anesthetized animals using a syringe with a 21G needle for biochemical and hematological analysis. Blood samples of 0.5 ml were placed in Microvette^®^ tubes containing K3EDTA for hematological analysis. Serum was obtained from remaining blood, which was stored at −20°C until biochemical analysis.

#### 2.2.7 Euthanasia

The animals were euthanized according to the following scheme: on the 3rd week - 6 animals from group 1, 6 animals from group 2, 6 animals from group 3; on the 6th week - 6 animals from group 1, 6 animals from group 2, 6 animals from group 3; on the 9th week - 6 animals from group 1, 6 animals from group 2, 6 animals from group 4, 6 animals from group 5; on the 12th week - 6 animals from group 1, 6 animals from group 2, 6 animals from group 4 and 6 animals from group 5 (see group affiliation in Table 1).Animals were euthanized by anesthesia with Zoletil/Xylazine mixture followed by total blood sampling from the inferior vena cava (about 10 ml).

#### 2.2.8 Hematology

Blood samples of 0.5 ml were placed in Microvette^®^ tubes containing K3EDTA for hematological analysis. Hematological analysis was carried out using a Mythic 18 hematological analyzer with a veterinary program (C2 DIAGNOSTICS S.A., France).

#### 2.2.9 Serum chemistry

For clinical chemistry analysis, the blood samples (all remaining blood) was placed in a tube without anticoagulant. The blood was allowed to clot for 50 min at the room temperature and centrifuged (1,600 x g, 4°C, 15 min) for serum separation. Serum was immediately frozen (at –20°C) until assayed. The serum was analyzed with a SAPPHIRE 400 (Prestige 24i) automatic biochemical analyzer (Tokyo Boeki, Japan) using reagents from Randox Laboratories Ltd.

#### 2.2.10 Histology

Samples of organs and tissues were fixed in 10% neutral formalin solution for at least 48 h, then washed in running tap water, dehydrated in ascending alcohols, and embedded in paraffin. Paraffin sections 3–5 μm thick, stained with hematoxylin and eosin, were examined using conventional light microscopy on an AxioScopeA1 microscope (Carl Zeiss, Germany). To assess the severity of histological changes, a scoring scale was used (Breljak et al., 2015).

#### 2.2.11 Statistical analyses

The results are expressed as means ± standard error of mean (MEAN ± SEM). The numbers of rats (n) used in the experiments are given in figure legends. The significance of differences in multiple comparisons was determined using Kruskal–Wallis test. Additionally, to identify intergroup statistical differences, the Mann-Whitney *U*-test was performed. Significance level was determined at *p* ≤ 0.05.

## 3 Results

### 3.1 Microscopic analysis of urine

**FIGURE 1:**
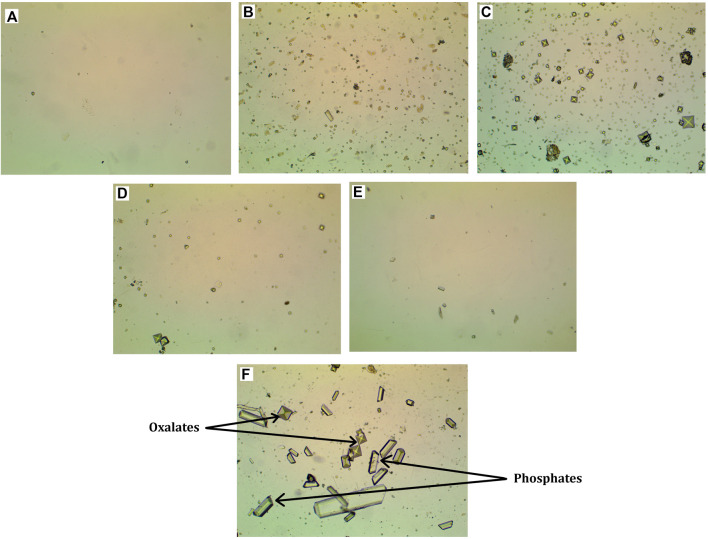
Micrographs of inorganic urine sediment. **(A)** - Control group, 3rd week of the experiment, **(B)** - Group EG 0.5%, 3rd week of the experiment, **(C)** - Group EG 0.5%, 6th week of the experiment, **(D)** - Group EG 0.5%, 9th week of the experiment, € - Group EG 0.5%, 12th week of the experiment, microscope magnification ×100, **(F)** - oxalic and phosphatic crystals, microscope magnification ×400.

Figure 1 shows photographs of the inorganic urine sediment of the control animal at week 3 of the study (A), as well as photographs of the inorganic urine sediment of the animal treated with EG 0.5% at weeks 3, 6, 9 and 12 of the study (B, C, D and E). The photographs show that in the EG 0.5% group, the amount of oxalates gradually decreases (Figures 1D,E). At week 6, oxalates of different sizes are present in the inorganic sediment (Figure 1C). By week 12 of the study, the number of crystals in the EG 0.5% group number of is comparable with the control animals (Table 2).

**TABLE 2:** The number of crystals in the inorganic urine sediment.

Group	Control (*n* = 6)	EG 0.5% (*n* = 6)	EG 0.5% + Ficus 1 (*n* = 6)	
3rd week				
oxalates	0.1 ± 0.1	44.4 ± 27.3**	18.1 ± 7.6*	
phosphates	41.5 ± 21.7	56.6 ± 21.0	43.1 ± 11.5	
6rd week				
oxalates	0 ± 0	41.2 ± 25.7*	15.8 ± 5.9**#	
Phosphates	39.3 ± 14.2	33.6 ± 10.2	45.7 ± 12.4**	
**Group**	**Control (*n* = 6)**	**EG 0.5% (*n* = 6)**	**EG 0.5% + Ficus 2 (*n* = 6)**	**EG 0.5%l + Cystone^®^ (*n* = 6)**
9th week				
oxalates	0 ± 0	1.1 ± 1.1	0.1 ± 0.1	0.1 ± 0.1
phosphates	35.4 ± 13.7	29.7 ± 17.0	29.3 ± 11.2	36.8 ± 12.9
12th week				
oxalates	0 ± 0	0.0 ± 0.0	0 ± 0	0.3 ± 0.3
phosphates	32.4 ± 20.6	36.1 ± 21.4	20.3 ± 8.9	32.7 ± 7.3

Data are presented as MEAN ± SEM.* - *p* < 0.05 comparing to control group according to Kruskal-Wallis test + Mann-Whitney *U*-test.

The crystals were counted as follows: the crystals were divided by size into three conditional categories: category 1 - crystals 9–17 μm in size, category 2 - crystals 5–7 μm in size, category 3 - crystals 2–4 μm in size. Then the average of the three categories for each group was found. In animals in which urolithiasis was modeled by drinking EG 0.5% instead of water for 6 weeks at the 3rd and 6th weeks of the study, the average amount of oxalates was significantly higher compared to control animals. Treatment with *Ficus tikoua* Bur. extract within 6 weeks of urolithiasis modeling led to a decrease in the amount of oxalates by 15% relative to the EG 0.5% group. At 9 and 12 weeks of the study, after the withdrawal of ethylene glycol, the amount of oxalates and phosphates between all groups did not differ.

### 3.2 Diuresis

After 2 and 6 weeks of EG 0.5% treatment diuresis was significantly reduced compared to the control group (Table 2). Application of *Ficus tikoua Bur.* extract during the first 3 weeks of urolithiasis modeling (EG 0.5% + Ficus 1) maintained of diuresis at the level of control animals. For 6 weeks of treatment with the extract of *Ficus tikoua* Bur. simultaneously with modeling of urolithiasis with EG 0.5%, diuresis was reduced relative to the control group, as well as in the group that received only EG 0.5%. On the 9th week of the experiment in the EG 0.5% group diuresis was lower than in the control and recovered to the control level by the 12th week (that is, 6 weeks after the withdrawal of EG 0.5%). Application of *Ficus tikoua* Bur. extract after urolithiasis modeling (EG group 0.5% + Ficus 2) as well as application of Cystone^®^, led to an increase in diuresis both in comparison with the control group and in comparison with the group with urolithiasis (Table 3).

**TABLE 3:** Diuresis results.

	Control (*n* = 6)	EG 0.5% (*n* = 6)	EG 0.5% + Ficus 1 (*n* = 6)	—
3rd week	67.8 ± 6	27.1 ± 2.8*	74.8 ± 24.8#	—
6th week	54.1 ± 8.2	27 ± 2.7*	30.2 ± 2.7*	—
**—**	**Control (*n* = 6)**	**EG 0.5% (*n* = 6)**	**EG 0.5% + Ficus 2 (*n* = 6)**	**EG 0.5% + Cystone^®^(*n* = 6)**
9th week	69.3 ± 7.9	29.4 ± 4.2*	110.5 ± 13.6*##	106.2 ± 26.3##
12th week	55 ± 10	42.1 ± 5.2	118.5 ± 11.4*#	127.9 ± 13.1**##

Data are presented as MEAN ± SEM; * - *p* < 0.05 comparing to control group according to Kruskal-Wallis test + Mann-Whitney *U*-test; # - *p* < 0.05 comparing to model (0.5% EG) according to Kruskal-Wallis test + Mann-Whitney *U*-test.

### 3.3 General urinalysis

At week 12 of the study, the use of Cystone^®^ led to a significant increase in the urine urobilinogen concentration by 73.9% relative to the control group. Application of *Ficus tikoua* Bur. extract had no effect on the urine urobilinogen concentration. It is worth noting that the level of urobilinogen was significantly increased in the Cystone^®^ group compared to the group that received *Ficustikoua* Bur. extract. Also in the group with *Ficus tikoua* Bur. extract and Cystone^®^ at week 12, an increase in pH was observed in comparison with the control group by 12.9%, and 14.3% relative to control, respectively (Table 4).

**TABLE 4:** General urinalysis results.

	Control (*n* = 6)	EG 0.5% (*n* = 6)	EG 0.5% + Ficus 2 (*n* = 6)	EG 0.5% + Cystone^®^(*n* = 6)
12th week				
Urobilinogen, mg/dl	7.3 ± 1.9	8.7 ± 1.6	6.7 ± 0.8#	12.7 ± 2.2*#&
pH	7.7 ± 0.3	7.7 ± 0.3	8.7 ± 0.3*	8.8 ± 0.1*

Data are presented as MEAN ± SEM.* - p < 0,05 comparing to control group according to Kruskal-Wallis test + Mann-Whitney U-test; # - p < 0,05 comparing to model (0.5% EG) according to Kruskal-Wallis test + Mann-Whitney U-test; &*- p < 0,05 comparing to EG, 0.5%+Ficus 2group according to Kruskal-Wallis test + Mann-Whitney U-test*.

### 3.4 Hematology

At the 3rd week of the study, in the EG 0.5% and EG 0.5% + Ficus1 groups, the percentage of lymphocytes was significantly increased relative to the control group. In the EG 0.5% group, the concentration of erythrocytes, the level of hemoglobin and hematocrit increased compared to the control group, and the cell volume and level of hemoglobin were reduced. RBC distribution width and standard deviation of RBC distribution width were significantly reduced after 3 weeks in all EG 0.5% groups. 3 weeks after the treatment with *Ficus tikoua Bur.* extract during the modeling of urolithiasis, the percentage of granulocytes was increased relative to the control group, and the level of lymphocytes normalized to the values of the control group. In the EG 0.5% + Ficus1 group, the concentration of erythrocytes was also increased compared to the control group on the 3rd week of the experiment, and hematocrit and hemoglobin were at the level of the animals from the control group and have statistically significant differences compared to the EG 0.5% group. At the 6th week of the study, in the EG 0.5% group deviations in hematological parameters were not found. The platelet distribution width was significantly increased compared to the control in the *Ficus tikoua* Bur*.* extract group at 9 and 12 weeks of the experiment. The erythrocyte level, hematocrit and hemoglobin were normalized after 6 weeks of *Ficus tikoua* Bur. extract treatment at week 12 of the study. On the 9th week of the experiment, in all groups taking EG 0.5%, the average concentration of hemoglobin was reduced. By the 9th week of the study, the use of the *Ficus tikoua* Bur*.* extract led to an increase in granulocytes relative to the control group. The level of erythrocytes in the group using *Ficus tikoua* Bur*.* extract was increased compared to the control group and on the 12th week of the experiment it normalized the percentage of granulocytes, while the use of Cystone^®^ for 6 weeks did not normalize the percentage of granulocytes (Table 5).

**TABLE 5:** Hematology parameters.

	Control (*n* = 6)	EG 0.5% (*n* = 6)	EG 0.5% + Ficus 1 (*n* = 6)
3rd week			
Lymphocytes, %	83.6 ± 0.9	83.7 ± 1.4	73.6 ± 4.3*#
Granulocytes, %	11 ± 1	12 ± 1	19 ± 5*
Red blood cells count (RBC), 10^12/L	6.9 ± 0.18	8.14 ± 0.12**	7.89 ± 0.15*
Haemoglobin (Hb), g/l	136 ± 2	151 ± 1**	142 ± 2#
Haematocrit (HCT), l/l	0.421 ± 0.006	0.472 ± 0.004*	0.446 ± 0.009#
Mean cell volume, fl	61.1 ± 0.7	58 ± 0.5*	56.2 ± 1.1*
Mean cell hemoglobin, pg	19.8 ± 0.3	18.6 ± 0.1	17.8 ± 0.3*#
RBC distribution width, %	12.5 ± 0.2	11.5 ± 0.2*	11.8 ± 0.2*
RDW standart deviation, fl	37.4 ± 0.6	8.14 ± 0.12**	8.14 ± 0.12**
Platelet count (PLT), 10^9/L	761 ± 17	695 ± 23	671 ± 34*
Mean platelet volume, fl	5.4 ± 0.1	5.4 ± 0.1	5.3 ± 0.1
Platelet distribution width, %	11.1 ± 0.3	13.8 ± 0.3*	14.2 ± 0.6*
6th week			
Lymphocytes, %	82 ± 1.3	78.7 ± 2	82.7 ± 1.3
Granulocytes, %	12 ± 1	16 ± 2	13 ± 1
Red blood cells count (RBC), 10^12/L	7.09 ± 0.1	7.74 ± 0.47	8.27 ± 0.19*
Haemoglobin (Hb), g/l	138 ± 1	140 ± 2	149 ± 3*
Haematocrit (HCT), l/l	0.431 ± 0.006	0.44 ± 0.01	0.472 ± 0.012*
Mean cell volume, fl	60.7 ± 0.9	57.6 ± 2.2	57.1 ± 0.8
Mean cell hemoglobin, pg	19.4 ± 0.2	18.4 ± 0.8	18 ± 0.3*
RBC distribution width, %	12.4 ± 0.3	12.5 ± 0.3	11.6 ± 0.2
RDW standart deviation, fl	36.5 ± 0.7	24.4 ± 7.7	26.5 ± 5.4
Platelet count (PLT), 10^9/L	741 ± 12	732 ± 12	690 ± 25
Mean platelet volume, fl	5.1 ± 0	5.3 ± 0.1	5.3 ± 0*
Platelet distribution width, %	10.9 ± 0.2	12.6 ± 1.3	14 ± 0.4*

	Control (*n* = 6)	EG 0.5% (*n* = 6)	EG 0.5% + Ficus 2 (*n* = 6)	EG 0.5% + Cystone^®^ (*n* = 6)
9th week				
Lymphocytes, %	79.8 ± 1.8	76.4 ± 1.9	74.9 ± 1.4*	77.3 ± 2.1
Granulocytes, %	13 ± 1	18 ± 2	20 ± 2**	17 ± 2
Red blood cells count (RBC), 10^12/L	8.34 ± 0.15	8.66 ± 0.11	8.85 ± 0.14	9.04 ± 0.13*
Haemoglobin (Hb), g/l	144 ± 4	144 ± 2	148 ± 3	149 ± 2
Haematocrit (HCT), l/l	0.459 ± 0.013	0.464 ± 0.009	0.479 ± 0.01	0.478 ± 0.006
Mean cell volume, fl	56.5 ± 1.1	53.5 ± 0.8*	54.1 ± 0.8	52.9 ± 0.8*
Mean cell hemoglobin, pg	17.7 ± 0.3	16.6 ± 0.2*	16.7 ± 0.2*	16.5 ± 0.2*
RBC distribution width, %	12.1 ± 0.2	12.4 ± 0.2	12.2 ± 0.1	12.5 ± 0.1
RDW standart deviation, fl	30.7 ± 0.1	30.6 ± 0.7	30.9 ± 0.4	31 ± 0.5
Platelet count (PLT), 10^9/L	658 ± 30	724 ± 17*	646 ± 12#	738 ± 25*
Mean platelet volume, fl	5.5 ± 0.1	5.4 ± 0	5.4 ± 0.1	5.5 ± 0.1
Platelet distribution width, %	14.3 ± 0.5	15.4 ± 0.2	14.8 ± 0.3	15.3 ± 0.6
12th week				
Lymphocytes, %	73.9 ± 2	69.1 ± 2.7	75.6 ± 2.7	70.7 ± 0.9
Granulocytes, %	21 ± 2	28 ± 4	23 ± 1	27 ± 1
Red blood cells count (RBC), 10^12/L	8.26 ± 0.17	8.43 ± 0.11	9.08 ± 0.35	8.63 ± 0.2
Haemoglobin (Hb), g/l	138 ± 2	142 ± 3	150 ± 4	139 ± 2
Haematocrit (HCT), l/l	0.44 ± 0.007	0.452 ± 0.009	0.494 ± 0.024	0.452 ± 0.008
Mean cell volume, fl	53.3 ± 0.5	53.6 ± 1.1	54.4 ± 1	52.5 ± 0.8
Mean cell hemoglobin, pg	16.7 ± 0.2	16.8 ± 0.4	16.8 ± 0.2	16.1 ± 0.3*#
RBC distribution width, %	12.1 ± 0.2	12.6 ± 0.3	12.3 ± 0.1	12.5 ± 0.1
RDW standart deviation, fl	29.8 ± 0.6	30.9 ± 0.8	31.3 ± 0.5	31 ± 0.8
Platelet count (PLT), 10^9/L	677 ± 18	687 ± 30	681 ± 29	680 ± 22
Mean platelet volume, fl	5.5 ± 0.1	5.4 ± 0.1	5.4 ± 0.1	5.5 ± 0.1
Platelet distribution width, %	15.2 ± 0.4	14.8 ± 0.6	15.6 ± 0.6	15.3 ± 1

Data are presented as MEAN ± SEM; * - p < 0.05 comparing to control group according to Kruskal-Wallis test + Mann-Whitney U-test; # - p < 0.05 comparing to model (0.5% EG) according to Kruskal-Wallis test + Mann-Whitney U-test.

Data are presented as MEAN ± SEM; * - *p* < 0.05 comparing to control group according to Kruskal-Wallis test + Mann-Whitney *U*-test; # - *p* < 0.05 comparing to model (0.5% EG) according to Kruskal-Wallis test + Mann-Whitney *U*-test.

### 3.5 Serum chemistry

A decrease in triglyceride concentrations was observed in all groups in which urolithiasis was modeled, regardless of the drug taken, throughout the study (Table 6). Modeling of urolithiasis led to a decrease in the concentration of chloride ion at the 3rd week of the studyin all animals treated with EG 0.5% chloride ions were significantly reduced compared to the control group by 2.4% in EG 0.5% group and by 2.5% in EG 0.5% + Ficus 1 group (Table 6). At week 9 of the study, chloride ion levels were reduced in the urolithiasis simulation group. At other time points in the study, no differences in chloride ion concentration were observed between groups (Table 6). At week 9 urea was significantly increased in all animals that consumed EG 0.5% compared to control.At week 12 of the study, inorganic phosphates were significantly reduced in the group EG 0.5%by 18.5%. Cystone^®^ and *Ficus tikoua* Bur. extract treatment resulted in a significant increase in the concentration of inorganic phosphates compared to the urolithiasis model (EG 0.5%), which is probably due to the increase of diuresis to 12th week. In the urolithiasis modeling group and in the Cystone^®^ group, a significant reduction in cholesterol levels was observed compared to the control. The use of *Ficus tikoua* Bur. extract, but not Cystone^®^, led to the normalization of cholesterol levels by the week 12.

**TABLE 6:** Serum chemistry parameters.

	Control (*n* = 6)	EG 0.5% (*n* = 6)	EG 0.5% + Ficus 1 (*n* = 6)
3rd week			
Urea, mmol/L	8.9 ± 0.3	8.5 ± 0.2	8 ± 0.6
Total Cholesterol, mmol/L	2.51 ± 0.17	2.56 ± 0.14	2.35 ± 0.15
Triglycerides, mmol/L	0.74 ± 0.14	0.96 ± 0.05	1.04 ± 0.19
Alanine aminotransferase (ALT), U/L	65 ± 7	78 ± 3	66 ± 8
Aspartate aminotransferase (AST), U/L	88 ± 6	81 ± 2	89 ± 5
Alkaline phosphatase (ALΡ), U/L	183 ± 21	220 ± 10	180 ± 18
Inorganic phosphates, mmol/L	2.24 ± 0.04	2.23 ± 0.05	2.35 ± 0.19
Na+	140.2 ± 1.1	138.5 ± 0.2	138.3 ± 0.8
Clˉ	98.6 ± 1.0	96.2 ± 0.3*	96.1 ± 0.6*
Globulin, g/L	23.6 ± 1.0	22.6 ± 0.6	22 ± 0.4
6th week			
Urea, mmol/L	8.1 ± 0.1	8.2 ± 0.1	8.3 ± 0.2
Total Cholesterol, mmol/L	2.67 ± 0.14	2.5 ± 0.11	2.63 ± 0.11
Triglycerides, mmol/L	0.82 ± 0.07	0.49 ± 0.04*	0.45 ± 0.04**
Alanine aminotransferase (ALT), U/L	80 ± 6	82 ± 4	84 ± 4
Aspartate aminotransferase (AST), U/L	113 ± 8	102 ± 7	111 ± 7
Alkaline phosphatase (ALΡ), U/L	164 ± 21	160 ± 11	160 ± 26
Inorganic phosphates, mmol/L	2.52 ± 0.11	2.51 ± 0.17	2.41 ± 0.13
Na+	142.4 ± 0.7	140.4 ± 0.6	141.6 ± 0.2
Clˉ	99.5 ± 0.7	98.4 ± 0.5	99.9 ± 0.5
Globulin, g/L	23 ± 0.6	22.4 ± 0.7	22.8 ± 0.5

	Control (*n* = 6)	EG 0.5%, (*n* = 6)	EG 0.5% + Ficus 2, (*n* = 6)	EG 0.5% + Cystone^®^, (*n* = 6)
9th week				
Urea, mmol/L	8.4 ± 0.5	11.9 ± 0.7*	12.0 ± 0.5*	11.0 ± 0.6*
Total Cholesterol, mmol/L	2.55 ± 0.19	2.64 ± 0.13	2.35 ± 0.12	2.60 ± 0.19
Triglycerides, mmol/L	1.23 ± 0.16	0.82 ± 0.06	0.67 ± 0.06**	0.76 ± 0.07*
Alanine aminotransferase (ALT), U/L	78 ± 3	75 ± 7	78 ± 5	77 ± 7
Aspartate aminotransferase (AST), U/L	78 ± 6	83 ± 4	80 ± 4	77 ± 4
Alkaline phosphatase (ALΡ), U/L	128 ± 28	149 ± 13	138 ± 10	134 ± 5
Inorganic phosphates, mmol/L	2.08 ± 0.08	2.07 ± 0.06	2.02 ± 0.08	2.09 ± 0.06
Na+	138.9 ± 0.3	139.2 ± 0.5	139.7 ± 0.6	140.1 ± 0.6
Clˉ	99.0 ± 0.2	97.9 ± 0.2*	99.8 ± 0.5	98.6 ± 0.4
Globulin, g/L	23.3 ± 0.3	25.2 ± 0.6*	22.7 ± 0.7#	24.1 ± 0.5
12th week				
Urea, mmol/L	9.4 ± 0.5	8.9 ± 0.5	9.7 ± 0.5	8.9 ± 0.3
Total Cholesterol, mmol/L	2.5 ± 0.11	2.19 ± 0.21*	2.92 ± 0.14#	2.34 ± 0.11*
Triglycerides, mmol/L	0.60 ± 0.07	0.45 ± 0.04*	0.48 ± 0.09*	0.41 ± 0.03*
Alanine aminotransferase (ALT), U/L	64 ± 7	65 ± 9	55 ± 15	60 ± 8
Aspartate aminotransferase (AST), U/L	97 ± 8	94 ± 3	97 ± 8	96 ± 5
Alkaline phosphatase (ALΡ), U/L	128 ± 29	144 ± 27	126 ± 25	154 ± 32
Inorganic phosphates, mmol/L	2.37 ± 0.10	2.00 ± 0.06*	2.57 ± 0.30	2.47 ± 0.08#
Na+	142.3 ± 1.1	141.1 ± 0.5	139.1 ± 1.5	141.2 ± 0.4
Clˉ	101.3 ± 1	98.8 ± 0.1	98.1 ± 0.7	99.5 ± 0.5
Globulin, g/L	25.0 ± 1.4	25.7 ± 1.3	24.1 ± 1.2	24.7 ± 1.1

Data are presented as MEAN ± SEM; * - p < 0.05 comparing to control group according to Kruskal-Wallis test + Mann-Whitney U-test; # - p < 0.05 comparing to model (0.5% EG) according to Kruskal-Wallis test + Mann-Whitney U-test.

Data are presented as MEAN ± SEM; * - *p* < 0.05 comparing to control group according to Kruskal-Wallis test + Mann-Whitney *U*-test; # - *p* < 0.05 comparing to model (0.5% EG) according to Kruskal-Wallis test + Mann-Whitney *U*-test.

### 3.6 Histological analysis

**FIGURE 2:**
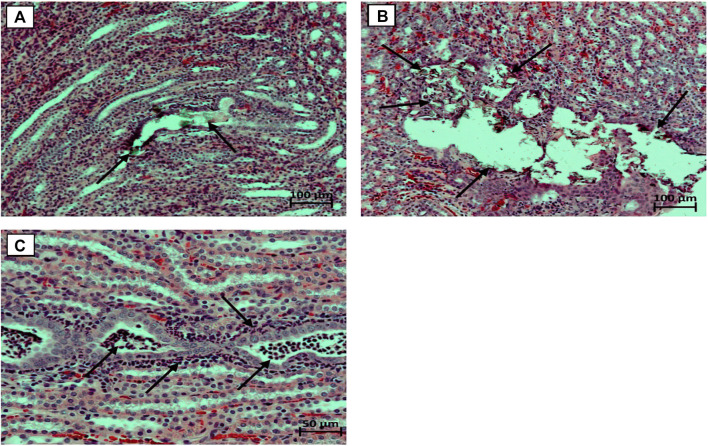
Micrographs of the kidneys. **(A)** - concrements in the lumen of the dilated renal tubule (marked with arrows), stained with hematoxylin and eosin, microscope magnification ×100; **(B)** - Randall plaque with multiple concrements in the region of the renal papillae of the pelvicalyceal system; stained with hematoxylin and eosin, microscope magnification ×100; **(C)** - Numerous segmented neutrophils in the lumen of the renal tubules, abundant peritubular neutrophil infiltration, stained with hematoxylin and eosin, microscope magnification ×200.

By the end of the 3rd week of exposure to EG 0.5% solution instead of standard drinking water, male rats showed initial signs of dilatation of the renal tubules without the presence of calculi in their lumen, in the pyelocaliceal system, in the lumen of the ureters and bladder. In some animals hyaline masses were found in the lumen of single dilated renal tubules, focal vacuolization of the bladder urothelium was noted. No differences in the pathomorphological picture in the kidneys among animals treated with saline and *Ficus tikoua* Bur. extractwere found. By the end of the 6th week, in the animals of the EG 0.5% group, a slight focal deformity of the renal corpuscles was observed in the cortical substance of the kidneys with the expansion of the lumen of the capsule and thickening of the outer leaflet, and proliferation of mesangial cells. The proximal and distal tubules were dilated, in isolated cases with the presence of calculi in their lumen (Figure 2A). In the epitheliocytes of the proximal and distal tubules, as well as in the collecting ducts of the medulla outer zone, pronounced signs of combined dystrophic changes were observed in the form of hyaline-droplet and (less often) hydropic dystrophy. At the same time, stones in the lumen of the ureters and in the cavity of the bladder were still not detected.In one animal treated with *Ficus tikoua* Bur. extract, numerous calculi were found in the pyelocaliceal system and in the lumen of the renal tubules with moderate focal dilatation of the renal tubules and the presence of Randall’s plaque (Figure 2B). Numerous segmented neutrophils were found in the lumen of single renal tubules in this animal; abundant neutrophilic infiltration was also noted around such tubules (Figure 2C).Thus, by the end of the 6th week of the study, a typical pathohistological picture is formed in the kidneys, which is characteristic of urolithiasis, while the use of *Ficustikoua*Bur. extract from the 1st day of the animals’ stay on EG 0.5% did not show any advantages relative to the background course of the pathologicalprocess.

**FIGURE 3:**
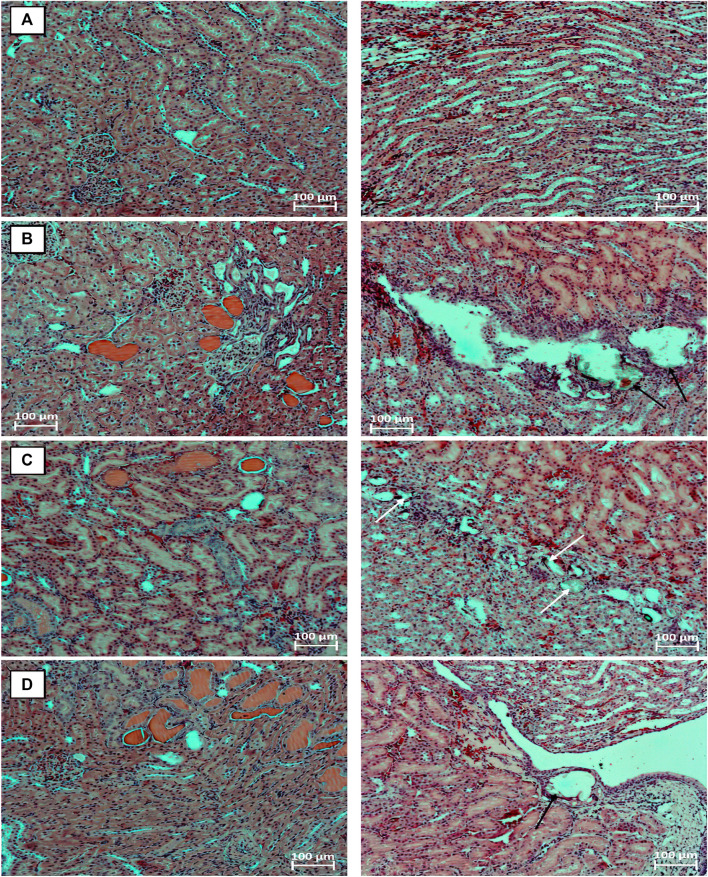
Fragments of the kidneys of intact animal **(A)**, animal with the model of urolithiasis by the end of the 12th week after a 6-weeks stay on aEG 0.5% solution instead of standard drinking water. Animals received from the 7th week water **(B)**, *Ficus tikoua* Bur. extract **(C)** and Cystone^®^ **(D)**. Phenomena of chronic progressive nephropathy with the presence of hyaline masses (pink) in the lumen of the dilated renal tubules; Randall’s plaques (black arrows); stones in the lumen of dilated renal tubules (white arrows). Stained with hematoxylin and eosin. microscope magnification ×100.

By the end of the 9th week, in animals of the EG 0.5% group with a background course of the pathologicalprocess, in four cases, minimal symptoms of chronic progressive nephropathy were found in combination with the presence of hyaline masses in the lumen of a few dilated renal tubules.A slight dilatation of the cavity of the renal tubules and focal degenerative changes in the epithelial cells of the distal renal tubules were observed in two animals of this group, in a single case, pronounced diffuse inclusions of lipofuscin were found in the cytoplasm of the epithelial cells of the renal tubules.No abnormalities were observed in the bladder and ureters.In the EG 0.5% + Ficus2 group, Randall’s plaque with slight dilatation of the pelvis was found in one animal. Focal degenerative changes in the epithelial cells of the renal tubules occurred in three cases, signs of chronic progressive nephropathy and the presence of hyaline masses in the lumen of the dilated renal tubules were observed in two cases. Among the animals treated orally with Cystone^®^ at a dose of 250 mg/kg from the day 42 of the study, stones in the pelvicalyceal system and renal tubules, as well as Randall’s plaques, were not found.At the same time, dilatation of the renal tubules occurred in three males from this group. By the end of the 12th week of the study in the EG group, 0.5% Randall’s plaques were found in three animals, in half of the cases, mild or moderate signs of chronic progressive nephropathy were found in combination with the presence of hyaline masses in the lumen, as a rule, of numerous dilated renal tubules (Figure 3B). In isolated cases, focal interstitial mononuclear infiltration, focal inclusions of lipofuscin in the cytoplasm of renal tubular epitheliocytes were encountered. Focal vacuolization of the urothelium was observed in the bladder of six male rats.Nochangeswerefoundintheureters. Among the animals of the EG0.5% + Ficus2 group, no significant improvements in the morphofunctional state of the kidneys were noted - identical pathohistological changes occurred in the same proportions as in the background course of the pathological process. Moreover, calculi were observed in the lumen of the renal tubules in one male rat (Figure 3C), and Randall’s plaques were still observed in a quarter of the animals. Against the background of oral administration to male rats from the 42nd day of the study of the drug Cystone^®^ at a dose of 250 mg/kg in the kidneys, the phenomena of chronic progressive nephropathy and hyaline masses in the lumen of the dilated renal tubules were less common, however, Randall’s plaques were found in two animals (Figure 3D).

## 4 Discussion

The search for drugs based on plant components is currently a crutial task in the treatment of urolithiasis (Bagcı, 2000; Fabricant and Farnsworth, 2001; Muthu et al., 2006). *Ficustikoua* Bur. has a wide range of biological activities and has long been used in traditional folk medicine for the treatment of a wide range of human diseases (Park et al., 2001; Chiang et al., 2005; Taiwo et al., 2006; Guan et al., 2007; Damu et al., 2009; Ahmed and Urooj, 2010; Chen et al., 2010; Donfack et al., 2010; Muanda et al., 2010; Thakare et al., 2010; Wang et al., 2010; Wei et al., 2012).

*Ficustikoua* Bur. extract contains multiple ingredients that work synergistically during the treatment to the disease, including kaempferol, an important flavonoid which has a wide range of pharmacological activities, including antioxidant, anti-inflammatory, antibacterial, anticancer, cardioprotective, neuroprotective, antidiabetic, antiosteoporosis, anti-estrogen, anti-anxiety, analgesic and anti-allergic effects. The extract including quercein derivatives and kaempferol derivatives can also cause the dog urine volume increased, ureteral peristalsis frequency increased, upper pressure of cavity tube increased. The possible reason is that the flavonoids have the activities of inhibition of xanthine oxidase. Bergapten from *Ficus tikoua* Bur. has vasodilating effect, anti-tumor activities and ability of clearing active oxygen free radical. Amentoflavone from *Ficus tikoua* Bur extract can exert anti-inflammatory activity through inhibition of transcription factor NF-κB activation which can induce the generation of nitric oxide synthase. Among these components, flavonoids are the main components, which we believe that they are actually the main active components.

To simulate urolithiasis, we chose to drink animals with EG 0.5% instead of drinking water. The concentration of the damaging agent EG was chosen by us on the basis of a previous study, in which it was shown that higher concentrations of EG, namely EG 1%, have a general toxic systemic effect (up to death) on the experimental animals, which makes it difficult to isolate only urolithiasis, as an independent disease, and, accordingly, makes it difficult to determine the effectiveness of the studied therapeutic agents for the treatment of specific urolithiasis (Bervinova et al., 2022).

We have conducted a study of the effectiveness of *Ficus tikoua* Bur. extract at a dose of 240 mg/kg with two different modes of administration in a model of urolithiasis. Cystone^®^, a drug based on herbal components, used in the clinic for the treatment of urolithiasis, was chosen as the reference drug [14]. In this work, we focused on studying the effectiveness of *Ficus tikoua Bur*. extract at two different modes of administration: simultaneously with the modeling of urolithiasis from 1 to 6 weeks of the study (EG 0.5% + Ficus 1) and during the recovery period, after the EG 0.5% withdrawal from 7 to 12 weeks of the study (EG 0, 5% + Ficus2). Cystone^®^ was started to be administered after the administration of EG 0.5% was canceled - from the 7th to 12th week of the experiment (EG 0.5% + Cystone^®^), which is comparable with the experience of clinical use of the drug after the development of urolithiasis.

It was no coincidence that we decided to use EG 0.5% to simulate urolithiasis instead of higher concentrations of EG. The use of EG 1% leads to multiple organ failure. The toxic effects of 1 and 0.75% ethylene glycol can result in the violation of liver functions, the levels of ALT, AST, ALP increase in the blood serum, acute renal failure, which ultimately leads to high mortality of animals (Yamaguchi et al., 2005; Breljak et al., 2015).When using EG 0.5%, by the 3rd week of the study, we did not find stones in the lumen of the renal tubules, in the pyelocaliceal system, in the ureters and in the lumen of the bladder. Signs of chronic progressive nephropathy, which occurred in 4 out of 6 animals, as well as hyaline masses in the lumen of dilated renal tubules, which occurred in 5 cases, should be attributed to background changes in the kidneys, which occur in approximately 30–70% of male rats of comparable age.The use of EG 0.5% leads to the formation of oxalate crystals in the inorganic urine sediment by the 3rd week. Oxalates were found in the inorganic urine sediment for 6 weeks while taking EG 0.5%.

*Ficus tikoua* Bur. extract showed its effectiveness in reducing the amount of oxalates as early as 3 weeks of its use, and the effect was observed for 6 weeks. Probably, the decrease in the amount of oxalates is associated with an increase in urine pH by the 12th week of the study. EG 0.5% treatment leads to the formation of oxalate crystals in the inorganic urine sediment by the 3rd week. Oxalates were found in the inorganic urine sediment during all 6 weeks of taking EG 0.5%. *Ficus tikoua* Bur. extract showed its effectiveness in reducing the amount of oxalates as early as 3 weeks of its use, and the effect was observed for 6 weeks. Decrease in oxalates is likely associated with an increase in urine pH by the 12th week of the study. The use of Cystone^®^ for 6 weeks after modeling urolithiasis led to an increase in phosphate crystals, which is associated with an increase in pH in the urine. In *Ficus tikoua* Bur. group similarly the pH was increased and the amount of oxalates was reduced. At week 12 of the study, the use of Cystone^®^ resulted in a significant increase in the concentration of urobilinogen in the urine compared with the control group, while the use of the extract of *Ficus tikoua* Bur. did not lead to changes in the level of urobilinogen. In the Cystone^®^ group, the concentration of urobilinogen was increased due to a significant diuresis increase. Application of *Ficus tikoua* Bur. extract led to the normalization of the concentration of urobilinogen, which was not observed in the group with Cystone^®^.

The results of biochemical analysis in our study show that treatment with 0.5% EG for 6 weeks does not affect serum liver enzyme levels. That is, there is no toxic effect of EG 0.5% on the liver when taken instead of drinking water for 6 weeks. In addition, no changes in creatinine, uric acid, and calcium levels were observed in the modeling of urolithiasis, in contrast to other studies of the development of urolithiasis (Jayakumari and Rao, 2007; İlhan et al., 2014; Letavernier et al., 2016; Wang et al., 2020). In our study, we found that the use of EG 0.5% leads to a decrease in the concentration of chlorine and phosphate ions, which is typical for changes in the kidneys excretory function. In urolithiasis model we observed increase in the concentration of urea in the blood plasma, and the decrease in the concentration of chloride and phosphate ions in animals treated with ethylene glycol. The serum urea concentration increase in the EG 0.5% group is consistent with studies that used other chemical lithogenic agents such as vitamin D, sodium oxalate, EG 0.75% with a short exposure time (Jayakumari and Rao, 2007; İlhan et al., 2014; Letavernier et al., 2016; Wang et al., 2020). *Ficus tikoua* Bur. extract, as well as Cystone^®^, had no effect on the concentration of triglycerides when applied both during simulation and after simulation.Significant differences between the *Ficus tikoua* Bur. extract and Cystone^®^groups were found in cholesterol concentrations. The use of *Ficus tikoua* Bur. extract, unlike Cystone^®^, led to the restoration of cholesterol levels to control values.With respect to the concentration of globulins, the use of *Ficus tikoua* Bur. extract restored the concentration of globulin to the level of the control group at the 9th week of the experiment.

At week 6 of the study, histological analysis did not reveal the effectiveness of the *Ficus tikoua Bur*. extract. Histological examination of the kidneys also showed that administration of EG 0.5% in animals instead of standard drinking water for 3 weeks can potentially accelerate the progression of nephropathy, as evidenced by the presence of hyaline masses in the lumen of the tubules in five out of six animals. In the functional morphology of the kidneys, no significant favorable differences were found at the 9th and 12th weeks of the experiment among animals treated with *Ficus tikoua* Bur. extract from the 7th week of the study and Cystone^®^, compared with the use of *Ficus tikoua Bur*. extract during the formation of urolithiasis. Changes found in the histological examination when using *Ficus tikoua Bur*. extract in one animal did not entail significant changes in the biochemical profile of the entire experimental group.Thus, we cannot speak about the protective activity of *Ficustikoua Bur*.extract regarding the formation of calculi.

Diuresis decrease after 6 weeks of treatment with EG 0.5% in our study is consistent with the results reported by Patel et al., used 0.75% ethylene glycol for 2 weeks (Patel and Acharya, 2020). Application of *Ficus tikoua* Bur. extract and Cystone^®^ led to the restoration of diuresis at 3, 9 and 12 weeks of the study. Cystone^®^, in contrast to the *Ficus tikoua Bur*. extract, showed a significant increase of diuresis after 6 weeks of its use relative to the control value. Use of the *Ficus tikoua Bur*. extract did not lead to diuresis changes. Application of *Ficus tikoua* Bur. extract as well as Cystone^®^, restores diuresis during urolithiasis modeling, which is confirmed by the restoration of the concentration of inorganic phosphates, sodium and chlorine ions to the control level. *Ficus tikoua Bur*. extract treatment does not lead to significant deviations in diuresis, the content of urobilinogen and pH in the urine in contrast with Cystone^®^. The use of *Ficus tikoua* Bur. extract for 6 weeks during the modeling of urolithiasis reduced the level of oxalates in the inorganic urine sediment.

In hematological parameters, at week 3 of the study, the percentage of lymphocytes and granulocytes was increased relative to the control in groups treated with EG 0.5%. An increase in leukocytes, granulocytes and a decrease in hemoglobin are characteristic of the clinical picture of urolithiasis (Moyes et al., 2017). Treatment with EG 0.5% induced an increase in hemoglobin, in contrast to the opposite picture of urolithiasis in the clinic. The *Ficus tikoua* Bur. extract, which was administered for 3 weeks, normalized the percentage of lymphocytes and granulocytes, in contrast to Cystone^®^. The use of the *Ficus tikoua* Bur. extract, in contrast to Cystone^®^, led to the normalization of the percentage of granulocytes by the 12th week of the experiment. It can be concluded that the *Ficus tikoua Bur*. extract used both during the modeling of urolithiasis and during the recovery period, shows effectiveness in relation to the inflammatory process compared to Cystone^®^. Modeling of urolithiasis using EG 0.5% led to an increase in hematocrit in the whole blood of animals on the 3rd week of the experiment, and daily intake of *Ficus tikoua* Bur. extract against the background of modeling urolithiasis led to the restoration of hematocrit to the control level. As for other hematological parameters, *Ficus tikoua* Bur. extract did not affect the course of urolithiasis in animals receiving EG 0.5%. In contrast to clinical studies of hematological parameters in urolithiasis, in our study, the RBC distribution width and the RBC distribution width standard deviation the in all EG 0.5% treated groups were significantly reduced after 3 weeks, and the platelet distribution width was increased (Demiray et al., 2016). *Ficus tikoua* Bur. extract treatment both during the urolithiasis modeling and during the recovery period, decreased inflammatory process, unlike Cystone^®^.

*Ficus tikoua* Bur. extract can be used as an effective maintenance therapy from the initial stages of urolithiasis development.

## 5 Conclusion

*Ficus tikoua* Bur. extract application during urolithiasis modeling can potentially slow down the rate of chronic progressive nephropathy development. *Ficus tikoua* Bur. extract treatment both during the urolithiasis modeling and during the recovery period, decreased inflammatory process, unlike Cystone^®^. Application of *Ficus tikoua* Bur. extract as well as Cystone^®^, restores diuresis during urolithiasis modeling, which is confirmed by the restoration of the concentration of inorganic phosphates, sodium and chlorine ions to the control level. *Ficus tikoua* Bur. extract treatment does not lead to significant deviations in diuresis, the content of urobilinogen and pH in the urine in contrast with Cystone^®^. The use of *Ficus tikoua* Bur. extract for 6 weeks during the modeling of urolithiasis reduced the level of oxalates in the inorganic urine sediment. *Ficus tikoua* Bur. extract can be used as an effective maintenance therapy from the initial stages of urolithiasis development.

## Data Availability

The raw data supporting the conclusions of this article will be made available by the authors, without undue reservation.
